# Conceptual Design of a High-flux Multi-GeV Gamma-ray Spectrometer

**DOI:** 10.1038/s41598-020-66832-x

**Published:** 2020-06-18

**Authors:** K. Fleck, N. Cavanagh, G. Sarri

**Affiliations:** School of Mathematics and Physics, The Queen’s University of Belfast, BT7 1NN Belfast, United Kingdom

**Keywords:** Applied physics, Electronics, photonics and device physics, Particle physics

## Abstract

We present here a novel scheme for the high-resolution spectrometry of high-flux gamma-ray beams with energies per photon in the multi-GeV range. The spectrometer relies on the conversion of the gamma-ray photons into electron-positron pairs in a solid foil with high atomic number. The measured electron and positron spectra are then used to reconstruct the spectrum of the gamma-ray beam. The performance of the spectrometer has been numerically tested against the predicted photon spectra expected from non-linear Compton scattering in the proposed LUXE experiment, showing high fidelity in identifying distinctive features such as Compton edges and non-linearities.

## Introduction

High-energy gamma-ray beams are of central interest for a wide range of physical subjects, and present appealing characteristics for a series of practical applications. For instance, a wide range of astrophysical phenomena generate gamma-ray beams with energies spanning from a few MeV up to several TeV^1^. The understanding of high-energy gamma-ray astronomy is indeed one of the main routes towards a detailed understanding of high-energy astrophysical phenomena.

On a laboratory scale, brilliant sources of gamma-ray beams are an ideal tool to study nuclear phenomena (see, for instance, ref. ^2^) and to investigate fundamental quantum electrodynamic processes^3^. These sources are mainly produced by exploiting bremsstrahlung radiation resulting from the propagation of ultra-relativistic electron beams through a high-Z solid target (see, for instance, refs. ^4–6^) or via incoherent Compton scattering of an electron beam through the focus of an intense laser^7,8^. Other mechanisms, exploiting the near-term generation of multi-PW laser facilities, include direct laser irradiation of solids^9,10^, or electromagnetic cascades^11^. Conversion efficiencies from laser to gamma-ray photons exceeding 10% can be achieved with the aforementioned methods.

High-power laser systems are also opening up the possibility of studying high-field quantum electrodynamics in a controlled laboratory environment. Exotic phenomena such as quantum radiation reaction^12,13^, stochastic photon emission^14^, and pair production and cascading in a laser field^15,16^ are now experimentally accessible. Several large-scale facilities and experimental campaigns are currently being explored, including the LUXE experiment at the Eu-XFEL^17^ and the E-320 experiment at FACET-II, following the first seminal experiment in the area carried out at SLAC^18,19^. These processes are accompanied by the emission of ultra-short, high-brilliance, and high-energy gamma-ray beams^7,8,12^. Measuring the spectrum of these photons is expected to provide precious information about the behaviour of particles interacting with ultra-high fields.

Providing on-shot, detailed spectral measurements of high-brightness and high-energy gamma-ray beams is thus highly desirable for the progress of these research areas. Different systems have been proposed: methods based on pair production in a high-Z target^4,20,21^ are able to detect high-energy photons but are currently not designed to work at a high flux. Similarly, methods based on measuring the transverse and longitudinal extent of cascading in a material are designed to work only at a single-photon level, or present limited energy resolution for high fluxes^22^. Compton-based spectrometers (such as the one in ref. ^23^) do work at high-fluxes but can only meaningfully measure spectra up to photon energies of a few tens of MeV. Cherenkov radiation is also used^24^ but, again, it is best suited to perform single-particle detection. Large-scale detectors such as the EUROBALL cluster^25^ and the AFRODITE germanium detector array^26^ can also resolve up to 10–20 MeV but their significant size make their implementation in many laboratories infeasible.

In this paper, we report on a design of a compact gamma-ray spectrometer, which can provide live and non-invasive information on the absolutely calibrated spectrum of high-energy (scalable from hundreds of MeV to tens of GeV) and high-flux gamma-ray beams. In a nutshell, the photons are converted into electron-positron pairs during propagation through a thin high-Z solid target. The measured spectra of the pairs generated are then used to reconstruct the primary gamma-ray beam. The minimum number of photons realistically detectable is of the order of 10^5^ photons/GeV/event and an energy resolution of the order of a few percent at 10 GeV can be achieved. The performance of the system is numerically tested for the expected Compton-scattered spectra from the LUXE experiment (see Fig. 7 in ref. ^17^).

## Interaction of multi-GeV photons with a high-Z material

In this article, we will consider a solid target with a thickness that is much smaller than its radiation length. In this case, the main process via which a multi-GeV photon interacts with a material is pair production in the nuclear field and multi-step cascades can be neglected.

**Figure 1:**
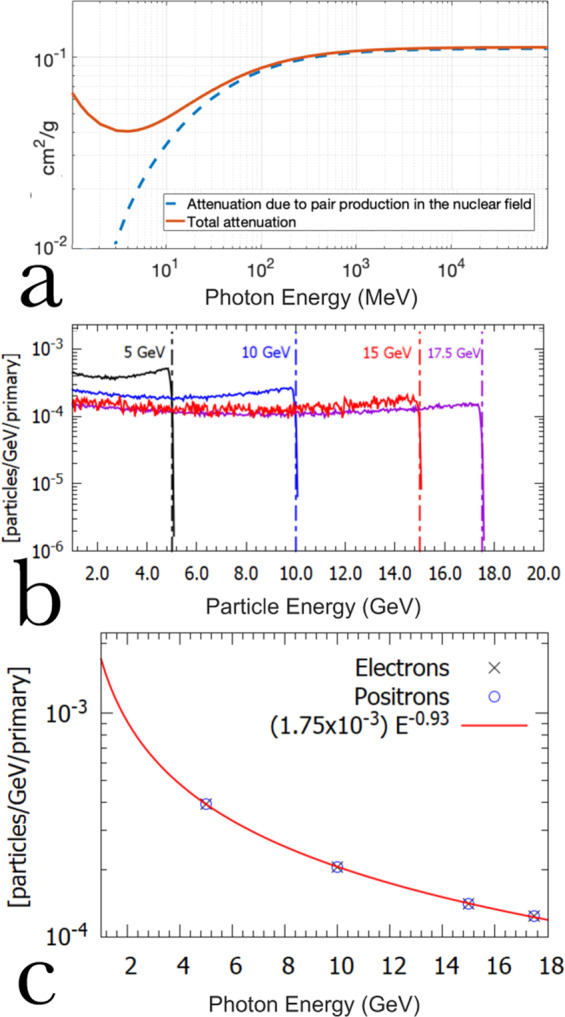
GeV-scale photon interaction with a thin tungsten target (**a**) Photon attenuation through tungsten as a function of energy: total (orange solid line) and due to pair production in the nuclear field (blue dashed line). Data taken from the NIST database^27^. (**b**) Simulated electron spectra (solid lines) at the rear surface of a 10 *μ*m tungsten foil irradiated by mono-energetic photon beams of different energies: 5, 10, 15, and 17.5 GeV (dashed lines). (**c**) Number of electrons/positrons per GeV per primary photon as a function of photon energy.

This is demonstrated by Fig. 1a, which shows the total attenuation and that due to pair production in the nuclear field of a photon through tungsten as a function of its energy^27^. Above 100 MeV, attenuation is entirely dominated by pair production. The total cross-section for pair production in the nuclear field can be expressed, in the ultra-relativistic approximation, as^28^:

1 $$\sigma \approx \frac{28}{9}\alpha {Z}^{2}{r}_{e}^{2}\left[\log \left(\frac{2{E}_{\gamma }}{{m}_{e}{c}^{2}}\right)-\frac{109}{42}\right],$$

where *α* ≈ 1/137 is the fine-structure constant, *Z* is the element atomic number, *r*_*e*_ ≈ 2.8 × 10^−13^ cm is the classical electron radius, *E*_*γ*_ is the photon energy, and *m*_*e*_*c*^2^ is the rest energy of the electron. There is only a weak logarithmic dependence on the photon energy, implying approximately the same amount of electron-positron pairs generated regardless of photon energy. For GeV-scale photon energies and a tungsten nucleus, Eq. 1 predicts a cross section of the order of *σ* ≈ 10^−23^ cm^2^. For a 10 micron thick solid converter, this results into a conversion of photons into pairs of the order of 0.1%. Moreover, the emitted electrons and positrons will present an almost flat spectral distribution. This is elucidated by Fig. 1b, which shows the electron and positron spectra generated during the propagation of a pencil-like photon beam of different energies through 10 *μ*m of tungsten. The data shown is obtained from Monte-Carlo simulations, using the code FLUKA^29^, where 10^7^ mono-energetic photons of different energies were made to interact with a 10 *μ*m thick tungsten foil.

Based on these simulations, we can then extract a dependence of the spectral distribution of pairs as a function of photon energy, as shown in Fig. 1c. The number of electrons/positrons per incoming photon per GeV can be expressed as: *N*_*e*_/GeV/primary ≈ 1.7 × 10^−3^*E*[GeV]^−0.93^. As expected, the number of pairs per energy interval scales as the inverse of the photon energy, in quantitative agreement with the estimates from Eq. 1. Choosing a different material will not change the power law dependence on energy but only the multiplying coefficient (i.e., 1.7 × 10^−3^ in this case of a 10 *μ*m thick tungsten target).

As described later, this dependence will be used to reconstruct the spectrum of the primary photon beam from the recorded spectra of electrons and positrons after the converter. As to what concerns the spatial distribution of the generated pairs, one can assume, in an ultra-relativistic regime and for thin converters, that their scattering inside the material be negligible. The divergence of the pairs *θ*_*e*_ at the exit of the converter target will thus be dominated by the initial divergence of the photon beam *θ*_*γ*_ and the cone-angle of the pair-production process, which is of the order of the inverse of the Lorentz factor (*γ*_*e*_) of the particle:

2 $${\theta }_{e}\approx \sqrt{{\theta }_{\gamma }^{2}+1/{\gamma }_{e}^{2}}$$

This relation is found to be in good agreement with the numerical simulations discussed below.

## Tracking and Signal-to-Noise considerations

**Figure 2:**
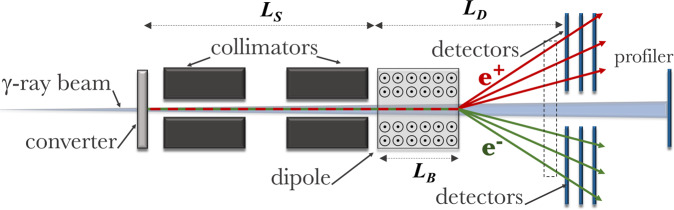
Sketch (not in scale) of the setup, highlighting the main constituents of the system. The dashed square represents the region where the transverse distribution of the particles, shown in Fig. 3c, is taken.

The main function of the spectrometer (sketched in Fig. 2) is thus to measure the spectrum of electron/positron pairs exiting the converter target, from which the spectrum of the primary gamma-ray photons can be reconstructed. To do this, a simple magnetic spectrometer consisting of a dipole magnet and two detector regions can be used. It is interesting to note that the pair production process effectively produces identical spectra of electrons and positrons. Measuring both simultaneously thus provides a useful consistency check of the system and, probably more importantly, allows one to efficiently identify noise sources in the system.

Particular care must be taken in optimising the signal-to-noise ratio (S/N). The main sources of noise in the system can be identified as: events involving an interaction with any component of the spectrometer other than the converter - such as dipole magnets and collimators -, off-axis photons and low-energy electron and positron pairs exiting the converter. These lower energy electrons and positrons (sub-GeV) will exit with broad divergences, and could thus be redirected by the dipole magnet onto the detectors. If we assume that we are interested in photon beams with energies exceeding the GeV, and that these beams will have relatively small divergences, i.e., of the order of a mrad, most of the GeV-scale electron and positron pairs will exit the converter with a similar divergence. We can then introduce high-Z, small-aperture long collimators to kill off-axis particles and photons and select only the high-energy part of the electron-positron pairs generated.

**Figure 3:**
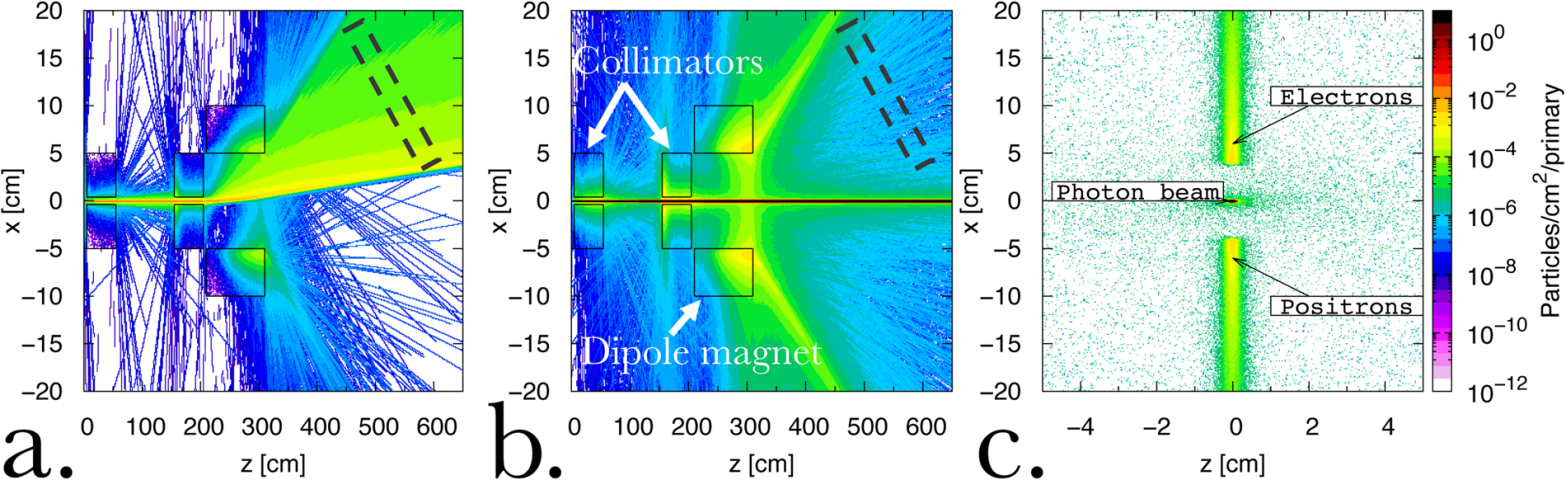
Simulated noise and signal spatial distribution in the spectrometer Electron (**a**) and photon (**b**) spatial distribution after entering the system sketched in Fig. 2. The positron and electron distributions are symmetrical around the longitudinal axis of the spectrometer (positron distribution not shown). Dashed rectangles indicate a possible position for the detector. (**c**) Corresponding transverse distribution of electrons, positrons, and photons at the back of the spectrometer (dashed rectangle in Fig. 2). Electrons and positrons are detectable with a signal-to-noise ratio larger than 10.

As an example, we show in Fig. 3 results from a FLUKA simulation of the propagation of a 15 GeV photon beam (10^7^ primaries initialised in the simulation) through the proposed spectrometer design sketched in Fig. 2. For the rest of the article, we assume the whole system to be in vacuum, to significantly reduce the computational cost of the simulations. While we acknowledge that running the whole system in vacuum might represent a significant experimental challenge, it is a preferable option also from an experimental point of view, since the system would not be susceptible to the interaction of the pairs with air, which would add complication to the data analysis and create an additional source of noise. In these simulations, we assumed a magnetic field of *B* = 0.5 T and the geometrical quantities defined in Fig. 2: *L*_*B*_ = 1 m, *L*_*S*_ = 2 m, and *L*_*D*_ = 4 m. Two 50-cm long collimators, one right after the collimator and one one meter away are assumed. They are both made of lead and have apertures on axis with a diameter of 8 mm, corresponding to an angular acceptance of 4 mrad. As shown in Fig. 3, they are effective in minimising off-axis noise from the converter, with only two spikes of noise corresponding to low energy particles hitting the dipole magnet frame (Fig. 3b). However, these spikes are outside the range of detection (dashed rectangles in Fig. 3) and can thus be ignored. A double-collimator system is to be preferred to a single collimator even though this increases the overall size of the spectrometer. This is because internal reflection of particles within the aperture of a single collimator would still represent a significant source of noise, which is well mitigated by the second collimator. In Fig. 3c we show the transverse distribution of electrons, positrons, and photons at the back plane of the system (shown as a dashed rectangle in Fig. 2). As one can see the spectrally dispersed positron and electron streaks are clearly detectable with S/N > 10 and a signal of ≈10^−4^–10^−3^ particles/primary photon/cm^2^.

## Reconstruction of the gamma-ray spectrum

Once the electron and positron spectra are recorded, the curves in Fig. 1b,c can be used to reconstruct the spectrum of the primary photon beam. In a nutshell, the number of electrons and positrons at the highest recorded energy is measured, and the number of photons responsible for that population of particles is extracted. Then, the spectrum of the electrons and positrons generated by these highest energy photons is subtracted from the original electron and positron spectra. The procedure is then repeated for progressively smaller photon energies. A critical quantity that has to be defined in this procedure is the size of the energy bin over which the estimation of the number of electrons and positrons is carried out. Intuitively, a small energy bin will result in higher energy resolution but will contain fewer particles. To identify the ideal energy bin size, one should then first consider what is the intrinsic energy resolution of the spectrometer. In the ultra-relativistic limit, this can be expressed as^30^:

3 $$\frac{\Delta \,{E}_{e}}{{E}_{e}}\simeq \frac{({L}_{S}+{L}_{D})}{c({L}_{D}-{L}_{B}/2){L}_{B}}\cdot \frac{{E}_{e}}{eB}\cdot {\theta }_{e},$$

where *L*_*S*_, *L*_*D*_, and *L*_*B*_ are geometrical quantities defined in Fig. 2, *E*_*e*_ is the particle energy, *B* is the magnetic field strength, and *θ*_*e*_ is the particle divergence at that energy. From Eq. 2, the divergence of the electrons and positrons is dictated by the divergence of the primary photon beam and the spreading induced by the pair-production process in the converter.

**Figure 4:**
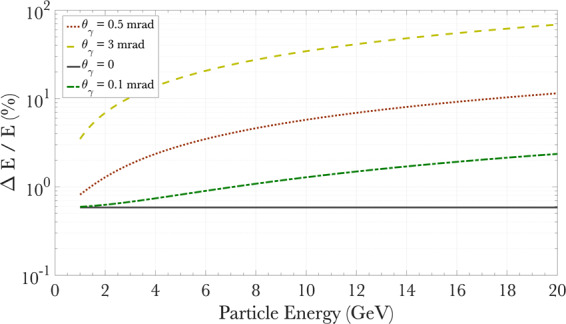
Energy-dependent spectral resolution of the spectrometer for different divergences of the primary photon beam.

For a 1 GeV photon, this spread is ≈1/*γ*_*e*_ ≤ 0.5 mrad (down to 50 *μ* rad at 10 GeV). As an example we plot, in Fig. 4, the energy-dependent resolution of the spectrometer for the parameters aforementioned. Neglecting for now the pixel size of the detectors, an ideally collimated photon beam (no divergence) could in principle be spectrally resolved with a relative uncertainty in energy of ≤1%. This results from the divergence (*θ*_*e*_ in Eq. 3) induced by the pair production process inside the converter. A more realistic photon beam divergence in the mrad range will result in spectral resolutions of the order of 10% at 10 GeV (1% at 1 GeV). It is thus not meaningful to choose energy bin sizes smaller than these quantities. In the example below, the bin size for the gamma-ray reconstruction is kept constant throughout the spectrum at 250 MeV. This is an idealised case to show the performance of the spectrometer, with the understanding that the energy binning size is strongly dependent on the specific setup to be adopted, as it is influenced, for instance, by the divergence of the photon beam to be measured and the physical size of the detectors’ pixels.

**Figure 5:**
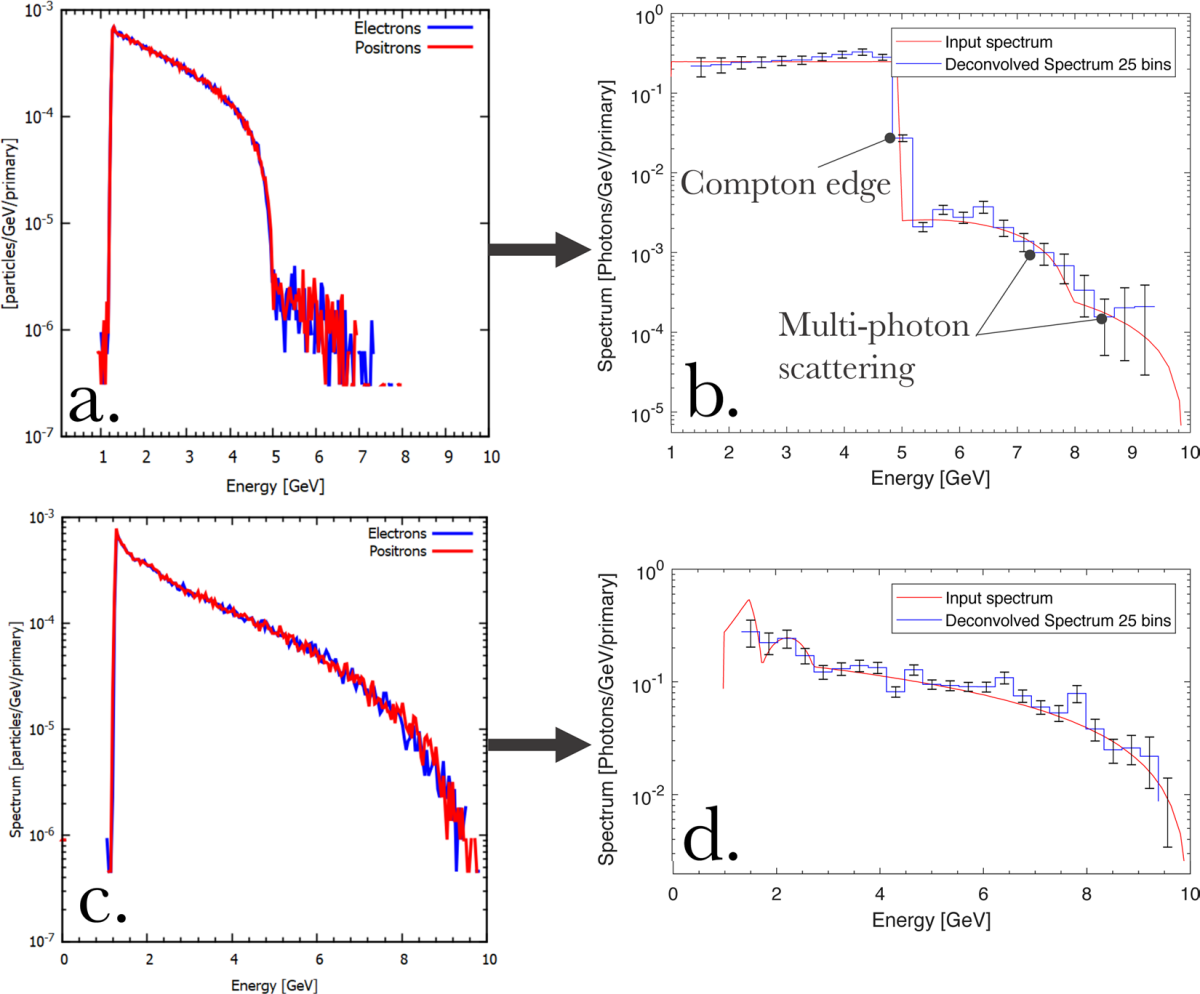
Example of reconstruction of the gamma-ray spectrum. (**a**,**c**) simulated electron and positron spectra at the detector plane for the incident gamma-ray spectrum shown as a red line in frame (**b**,**d**) (**b**,**d**) Comparison between the original input spectrum (red line) and the one predicted by the reconstruction algorithm (blue line) using the simulated electron and positron spectrum shown in frame (**a**,**c**). Distinctive features in the spectrum are highlighted in frame (**b**).

As an example of the effectiveness of the spectrometer, we show here the performance of the proposed design in measuring the structured spectra of photon beams expected from Compton scattering in the LUXE experiment (Fig. 7 in ref. ^17^), where the 17.5 GeV electron beam from the Eu-XFEL will be collided with a focussed laser pulse with a maximum dimensionless intensity of the order of ${a}_{0}\simeq 2$. We choose two cases: non-linear (*a*_0_ = 2) and linear (*a*_0_ = 0.2) Compton scattering spectra (Fig. 7 of ref. ^17^ and red lines in Fig. 5b,d, respectively). Photon beams with such spectra are sent, using the Monte-Carlo code FLUKA, through the system sketched in Fig. 2 and the resulting electron and positron spectra at the detector plane are recorded.

**Figure 6:**
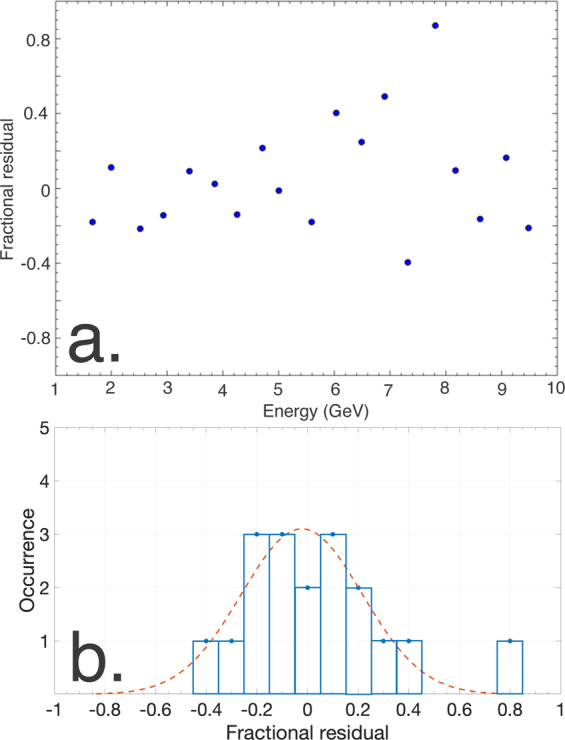
Accuracy in yield of the spectrometer (**a**) Relative difference between the input gamma-ray spectrum and the prediction of the reconstruction algorithm as a a function of energy for the case shown in Fig. 5b and a constant energy bin size of 250 MeV. (**b**) The distribution of the residuals is reasonably approximated by a Gaussian distribution with a standard deviation of 23%.

The reconstruction algorithm is then applied to these electron and positron spectra to retrieve the original gamma-ray spectrum. The results are shown in Fig. 5, where the electron and positron spectra obtained from FLUKA simulations are shown in Fig. 5a,c for the cases *a*_0_ = 2 and *a*_0_ = 0.2, respectively, and the corresponding predictions of the reconstruction algorithm are compared with the original gamma-ray spectra in Fig. 5b,d. In this example, the maximum divergence of the Compton-scattered photons is obtained in the non-linear regime (*a*_0_ > 1)^7,8^ and is of the order of *θ*_*γ*_ ≈ *a*_0_ · *m*_*e*_*c*^2^/*E*_*γ*_ ≈ 60 *μ*rad, corresponding to an energy resolution at the percent level (Fig. 4). The algorithm yields an accuracy in photon yield of the order of 20% (see Fig. 6). These values are mostly due to the reconstruction algorithm and the size of the energy bin considered, and should be integrated with the uncertainty resulting from the electron and positron detection systems. However, it is clear from Fig. 5 that the system is able to precisely identify distinctive features in the spectra, such as the linear Compton edge and the different levels of perturbative non-linear contributions (labelled in Fig. 5b).

It is to be intended that additional aspects, specific to the particular setup to be adopted, will also factor in determining the energy resolution of the system. For instance, care has to be taken in choosing the electron and positron detectors, since the detector pixel size, together with the divergence of the photon beam to be spectrally resolved, will constrain the energy resolution and the amount of signal detected per pixel. From Fig. 3c, one can see that approximately 10^−4^–10^−3^ particles/primary photon/cm^2^ will be incident on the detectors, implying 10^5^–10^6^ particles/cm^2^ for a realistic primary photon beam containing 10^9^ photons. If we assume the idealised case of a pencil-like photon beam, the spectrometer could in principle reach down to a resolution of the order of 1% (i.e., 100 MeV at 10 GeV, see Fig. 4). To guarantee this energy resolution at 10 GeV, one would need a pixel size along the dispersion axis of the spectrometer of approximately 500 *μ*m, easily attainable with modern scintillators (approximately 1.3 mm for the example of a 250 MeV energy binning). We can then assume a 20 cm × 2 cm detector with 500 *μ* m × 1 cm pixels (800 pixels in total). In this case, the detector will receive a measurable quantity of the order of 10^4^ particles per pixel. Due to the non-linear energy dispersion relation of the dipole magnet, it is however not necessary to keep a constant pixel size throughout the detector, with the possibility of having larger sizes at the low-energy end, thus reducing the overall number of pixels required.

As a final remark, the spectrometer design is virtually transparent to the gamma-ray beam (≥99% of the photons propagate unperturbed through the system), allowing for downstream profiling and calorimetry to be fielded simultaneously with the spectrometer (sketched in Fig. 2).

## Conclusions

In conclusions, we report on a conceptual design for a gamma-ray spectrometer, specifically designed for high-fluxes and high-energies. The system exploits the approximately flat spectral distribution of the electron/positron pairs generated during the propagation of the gamma-ray beam through a thin high-Z converter. A possible design is presented for the LUXE experiment, showing the capability of spectrally resolving gamma-ray beams in an energy range between 1 and 10 GeV, with a signal-to-noise exceeding 10, a spectral resolution of the order of a few percent, and an accuracy in predicting the gamma-ray yield of the order of 20%. It is proposed that similar setups could be used to spectrally resolve high-flux and high-energy gamma-ray beams in a compact configuration, yielding precious information in high-energy physics and ultra-high intensity laser experiments.
